# Gadamer, Habermas and Ricoeur: Toward a Hermeneutic Philosophy of Care

**DOI:** 10.1111/nup.70049

**Published:** 2025-10-21

**Authors:** José Ricardo C. M. Ayres

**Affiliations:** ^1^ Department of Preventive Medicine—Medical School University of São Paulo São Paulo São Paulo Brazil

**Keywords:** gadamer, habermas, health care, hermeneutics, practical philosophy, ricoeur

## Abstract

This essay builds upon the dialogue with Missel and Birkelund's reflections on the contributions of contemporary hermeneutics—particularly the work of Paul Ricoeur—to health research. While endorsing these authors’ central thesis, the present discussion seeks to extend the argument by advocating for hermeneutics’ relevance to clinical practice as well. Drawing on the ideas of Gadamer, Ricoeur, and Habermas, the text critiques the excessive hegemony of techno‐scientific knowledge over clinical practice and its harmful consequences for outcomes. It argues that revisiting Aristotle's distinction between theoria, techne, and praxis and fostering a genuine synergy among these spheres of rationality represents a potential hermeneutic contribution to the emancipatory reconstruction of health practices. The concept of Care is adopted as the philosophical horizon for this critical‐hermeneutic reconstruction.

## 1 A Dialogue With Missel and Birkelund

Missel and Birkelund published an important article in Nursing Philosophy titled ‘Ricoeur's Narrative Philosophy: A Source of Inspiration in Critical Hermeneutic Health Research.’ (Missel and Birkelund 2020). What stands out in the article, beyond the clear and engaging writing style, is the authors’ introduction of a still relatively underexplored contribution to health research discussions, especially in English‐speaking countries: hermeneutics as a philosophical framework, particularly the distinctive features this theoretical tradition takes on in Paul Ricoeur's thought. Even rarer is their presentation of a Ricoeur understood through the dialogue the French thinker establishes between Hans‐Georg Gadamer and Jürgen Habermas, exponents of two distinct philosophical traditions with many points of convergence—Gadamer was a decisive influence on Habermas’ academic trajectory—but also some significant divergences (Müller‐Doom 2016).

As the authors note, Paul Ricoeur is not a frequent reference in the social and human sciences applied to health, even less so is this Ricoeur who immerses the critical perspective into the narrative horizon of interpretation, establishing a fruitful dialogue between Gadamer and Habermas (Ricoeur 1993a). Yet, as they argue, this critical‐hermeneutic perspective is essential when it comes to recognizing the ideological biases permeating narratives produced in health practices and the contextual aspects that lend greater intelligibility to the lived world of healthcare recipients and their agents, the healthcare professionals. We can understand such biases on several levels, from those of a broad socio‐political nature, such as the culturally hegemonic ways of conceiving the body and illness in contemporary capitalist societies, to the interpretative bias resulting from the uncritical appropriation of paradigmatic forms of biomedical knowledge throughout its historical transformations, such as causal reductionism, based on the etiological approach of Henle‐Koch, or the generalization of statistical findings, based on Evidence‐Based Medicine, or even the assumption of an absolute biological determinism, based on Precision Medicine. Biases can also be shown in the common sense, when we observe patients applying to themselves diagnoses constructed based on information that goes viral on social networks or seeking in medicalization simplistic solutions to complex social and/or psycho‐emotional situations.

However, there is one statement in the article's concluding remarks that warrants reflection. The authors state:

‘If the vision of a more patient‐centred health service is to be realized, we need to obtain research‐based knowledge about the patients’ view of the life situation they find themselves in. This kind of knowledge, which is generated through narrative‐based research, cannot be directly implemented in practical processes in clinical practice, but it can provide a human insight that can indirectly improve knowledgeability and quality in practice.’

While it is true that the techno‐scientific domain of health practices tends to hinder a critical‐hermeneutic approach at the moment of care, I believe it is not impossible. On the contrary, although undoubtedly counter‐hegemonic, a hermeneutic approach, which is somehow already present at the moment of any clinical practice—though frequently untransparent, devaluated and impoverished—seems not only possible but necessary to overcome the crisis of legitimacy and even efficacy that has led to reconstructive proposals in recent years, such as Patient‐Centred Care, Narrative Health, Humanized Care, etc. (Ayres 2022)

This essay seeks to explore the possibilities of a productive convergence between the contributions of Ricoeur, Gadamer, and Habermas and a philosophy of health practices capable of driving reconstructive movements into the very moment of clinical practice.

## 2 Care, With a Capital C!

A terminological clarification is necessary at this point. As noted by Missel and Birkelund, Ricoeur and other contemporary hermeneutic thinkers assume that language is the foundation and possibility for making sense of the world we inhabit and share, albeit not always consciously and not always expressed in a linguistically structured form—as in the case of very young children or people with disabilities or neurodiversity, whose linguistic expression may be restricted, even though they always have a language that situates them and places them in relation to others (Gadamer 1998). Thus, seeking to share this meaning through words is a fundamental and perennial pursuit of existence. For this reason, I explicitly state that I will use the word Care with a capital C here to designate a philosophical‐conceptual demarcation of a reconstructive conception of health practices (Ayres 2004). I use the idea of reconstruction in the Habermasian sense of a method of social analysis and criticism that aims to identify the conditions of possibility of certain social phenomena, with the aim of understanding how they are formed and how they can be transformed in an emancipatory sense (Thompson 1995). In the case of Care, this emancipatory sense refers to identifying in which aspects our health practices can become more effective as a resource to provide well‐being and happiness to individuals and populations. In other words, the proposal of Care arises from health practices still overly bound to the instrumentalism of techno‐sciences but which, through the experience of their limitations, point to hermeneutics as a path to overcoming them.

How, then, can we move toward Care in the daily routine of healthcare practices? A first step is to acknowledge that health care does not consist solely of the application of techno‐scientific knowledge to the apprehension of morpho‐functional disorders. Although such knowledge is fundamental, when attention is centered exclusively on it there is a tendency to reduce people to objects (of knowledge and intervention) and, in doing so, lose sight of the concrete possibilities of understanding singular meanings and conditions, a comprehension necessary to foster effective intervention.

In their article, Missel and Birkelund highlight the dialogical nature of field research for the production of qualitative knowledge in health. Similarly, in various practice settings, it is only through genuinely dialogical interaction that techno‐scientific knowledge can be adequately applied to the uniqueness of each individual or situation. Gadamer, in one of his writings on health, draws attention to the fact that two discursive approaches overlap when we refer to a ‘case’ requiring health intervention (Gadamer 1996a). One refers to the case as a particular manifestation of a certain set of regularities composing a general rule (the discourse on the anatomic‐physio‐pathological manifestations of illness). The other refers to the case as a singular condition, lived and signified by individuals and communities who experience transformations in the flow of their lives (Canguilhem 1978), bringing worry, discomfort, suffering, pain, limitation, or even death.

Knowing the case in the techno‐scientific sense is essential to explain the morphological and/or functional processes related to illness or its risk and to seek some form of resolution. However, without considering the case in the second sense, as lived singularity, we face limitations in understanding the socio‐environmental and psychosocial determination of these processes and even greater limitations in transforming these conditions, resolving them, or at least finding more satisfactory ways of dealing with them.

This dialectic relationship between explanation and understanding brings us to Ricoeur's concept of the ‘hermeneutical circle.’ (Ricoeur 1993b). Of course, here we are not dealing with the relationship between the formal structure of a text's components and the meaning/reference of the text as a whole, but rather with a relationship between a structured, nomological discourse on anatomico‐physio‐pathological processes and the reference constituted by lived experience of illness—where this discourse primarily originates and from which are conformed intervention purposes. Ricoeur himself authorizes this mimesis between textual interpretation and clinical action by reminding us that human actions, always endowed with meanings and sense‐references, can be understood as a text opened for interpretation.

Given the centrality of techno‐scientific discourse in constructing the meaning of our health practices—at least in modern Western culture—it would be impossible to understand any experience of the health‐illness‐care processes without relying on this discursive structure. On the other hand, as noted above, the objectivist, analytical, fragmentary, and instrumental discourse of biomedical sciences (focused on means‐ends relationships and efficient causality) is insufficient to account for the singularity of lived experience. Or, in Gadamer's distinction, it provides explanatory references for cases as particularities of general laws but not for interpreting cases as singular experiences.

An experience, according to Gadamer (2006), is by definition something unrepeatable. It is unique, temporalized, contingent, and dependent on human judgments and values. Thus, we must seek in other types of discourse and knowledge elements to understand health‐illness‐care processes to produce Care. Knowledge that, as Gadamer points out, resides in the sphere of rationality Aristotle distinguished as *practical wisdom*.

## 3 Practical Wisdom and the Abilities of Reason

Gadamer identify contemporary philosophical hermeneutics as the type of rational activity Aristotle defined as *phronesis*, or practical wisdom. This definition represents an important difference between aspects of his philosophy and that of his master Plato. If Plato delineated the territory of reason solely as transcending the plane of sensations and doxa toward ideas referring to necessary, perennial, and universal relations, Aristotle saw this as only one aspect of reason's exercise—*episteme*, in the rational sphere of *theoria*. He argued that reason also expressed itself in other human capacities, characterizing two other spheres of reason's abilities: *techne*, where the human ability to produce useful artifacts for life through *poiesis* manifests, and the sphere of *praxis*, which refers neither to the pursuit of truth nor utility but to the pursuit of the Good Life, the just and happy life, or *eudaimonia*. Here, it is *phronesis*, or practical wisdom, that is set in motion by reason: the capacity to make virtuous choices in seeking happiness amid life's contingencies. (Gadamer 1996b)

For Aristotle, as for many of his time, these spheres could not be conceived as independent of one another. In his cosmological conception of existence, the good, the beautiful or useful, and the truth are mutually related and hierarchically ordered. *Theoria* occupied a superior plane because it pertained to things that necessarily are, that cannot be otherwise, transcending the finite and mutable nature of the sublunar sphere where humans dwell. It is this lower sphere that *techne* and *praxis* occupy, subject to human action and decisions—*proairesis*. But, according to Aristotle, precisely because they are subject to intervention, to the choices humans can make about them, the use of reason, specific kinds of it, becomes necessary both to *techne* and *praxis*. A reason whose guiding principle is not the pursuit of truth but of utility and virtue.

**Figure 1:**
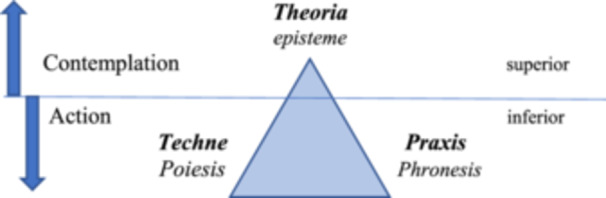
Spheres of Reason, Types of Knowledge, and Their Hierarchies According to Aristotle. *Source:* Author's creation.

We can graphically represent these spheres of rationality, their specific types of knowledge, and their hierarchy in Figure 1, as follows:

We know the profound transformations social life has undergone from Classical Antiquity to Modernity, with successive changes in modes of material production and reproduction. In line with these transformations the conceptions of reason and the hierarchies of its spheres have radically shifted. In modern times there is no longer room for a contemplative knowledge of Truth. Knowledge seeks to be, as Descartes stated in his famous Discourse on the Method, ‘most useful for life.’ The anthropocentric turn in modern culture, especially in the sciences, now seeks in the necessary relations of episteme motives and results that stem from human utilitarian interests and direct their products to them. And these necessary relations supporting the sciences no longer admit speculations transcending human senses and reasoning. Necessary relations are those logically and mathematically constructed, demonstrable in phenomena empirically verifiable by human senses and conceived with a view to their instrumental utility.

This does not mean that there are no contemplative components in the daily work of scientists, or that ideas of good and beauty are completely banished from their personal horizons. On the contrary, these aspects certainly play a role in the choices of theories and objects they make, in their heuristic speculations, and in the way they disseminate the products of their work (Bachelard 1987). What we want to point out is that, as a social process, scientific praxis becomes a central productive force in modern capitalist societies, and this makes its political, economic and institutional support intrinsically related to interests that are formed on its margins, in the sphere of technical development, exerting, in this sense, a strong influence on the formation and legitimization of scientific paradigms (Habermas 1971).

**Figure 2:**
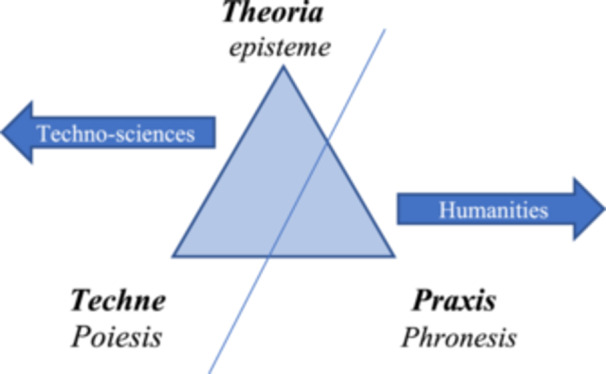
Modern Transformation of the Spheres of Reason, Types of Knowledge, and Their Hierarchies. *Source:* Author's creation.

Thus, we can say that the triangle in our scheme does not disappear, but its meanings and hierarchies certainly change, as shown in Figure 2:

Western health knowledge and practices follow this trend, becoming progressively oriented by the knowledge of positive sciences, especially those focusing on the biological basis of human existence. Since Claude Bernard and the development of his experimental pathology in the nineteenth century, health practices have become increasingly dominated by techno‐sciences ‐ in medicine and subsequently in other emerging fields such as nursing, dentistry, nutrition, physiotherapy, etc.

However, as known, the reasons people fall ill, how they interpret and deal with their health or lack thereof, their possibilities and interests in accessing care, and the diverse ways of doing so are not mechanically caused by physical‐chemical world relations or natural biological determinations. They are intrinsically linked precisely to that sphere of human experience tied to the contingent and to human choices and actions—the praxis.

For this reason, knowing and acting in the field of health, beyond their undeniable instrumental achievements, suffer harmful consequences from approaches overly centered on the relations between *episteme* and *techne*—the matrix of techno‐sciences—leading to the reconstructive reactions mentioned earlier, which many refer to as the pursuit of *humanization* of health practices.

**Figure 3:**
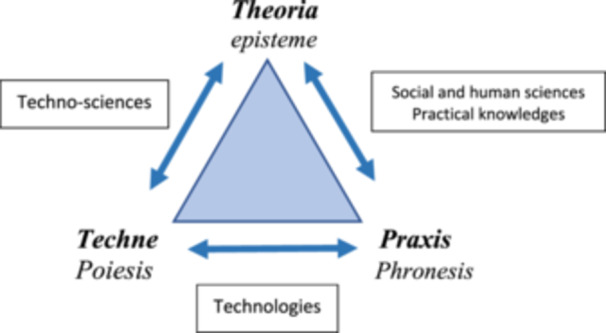
Relationships Between the Spheres of Reason from the Perspective of Care. *Source:* Author's creation.

Of course, the power of techno‐sciences for health protection and recovery is undeniable; we cannot renounce them. The problem is that without aligning this power with the questions of praxis, it becomes not only less effective in achieving its results but can also cause harm, as highlighted for example by discussions on quaternary prevention (Jamoulle 2015). Therefore, a new arrangement among the different spheres of rationality must be conceived—to which hermeneutics, this contemporary heiress of the Aristotelian practical philosophy, alerts us, as represented in Figure 3.

This arrangement points to the importance of continuous interrelation among the three spheres of human reason, ensuring technical interventions interact with both their scientific bases and the underlying practical interests, based on forms of validation—now using Habermasian terminology (Habermas 1991)—that combine propositional validity (statements accepted as true) with normative validity (those whose correctness—ethical, moral, political—is also accepted and meaningful to people).

It is also important to highlight that the aspect of normative validity in interactions between professionals and patients acquires peculiar and highly relevant characteristics when compared to other spheres of sociability. As Canguilhem (1978) points out, every health care is a technique for establishing and/or restoring a norm considered favorable to life that is constructed by experience, by our ways of living—norms that are always open, of course, to criticism and revision, but never ‘invented’ abstractly by the techno‐sciences of health. In this sense, it is life itself that conforms the categories of health and illness as normative judgments with which our techno‐sciences deal with. Therefore, as fruitless as prescribing or applying technical procedures in an authoritarian manner, without knowing and considering, through possible dialogue, the singularity of each patient and each therapeutic situation, will be to ignore the sense of establishing or maintaining norms favorable to life contained in some way in the techno‐sciences of health, neglecting their thoughtful and patient presentation to those we care for. Leaving the responsibility and consequences of their decisions regarding their health care exclusively to the people we care for, refraining from merging their existential horizons with those we hold as health professionals, is another way of evading the need to Care with a capital C. Hence the importance of producing the type of knowledge Missel and Birkelund discuss and ground in their text, but also of adopting the hermeneutic perspective of ‘fusion of horizons’ (Gadamer 2006) in the clinical practice, at the very moment of its realization. This is what we will address next.

## 4 Hermeneutics and Care

As noted earlier, the hegemony of techno‐sciences as the guiding knowledge of health practices tends to autonomize the technical and scientific poles in decision‐making about what to do and how to do it in response to health care demands, whether in prevention, treatment, or rehabilitation. But we know that any health intervention will always have practical implications in people's daily lives. The way we produce ‘health artifacts’ always reflects, in some way, what we consider adequate for the lifestyles we lead and the values we pursue. Technologies can thus be understood as socially legitimized modes (always amid tensions and contradictions) of generating, articulating, and applying techniques—with their material instruments and knowledge—for the production, conservation, and recovery of our ways of life. In our case, these are modes of health promotion, prevention, treatment, and recovery of ailments that align with the lifestyles of the people we care for.

Now, if in operating health technologies we forget this link between technique and praxis, emphasizing only the relations between techniques and their scientific validation, we turn the people and communities we care for into mere objects of our knowledge and interventions, negating their subjectivities, the practical meanings of their lives, their interests, their social contexts… in short, dehumanizing them.

**Figure 4:**
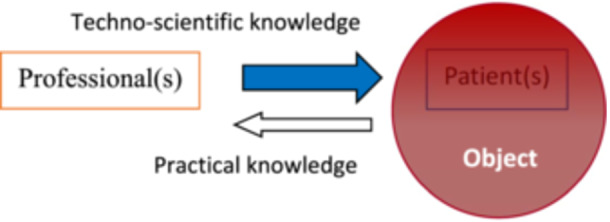
Technicist Interaction Model in Health Practices and Its dehumanizing Effect. *Source:* Author's creation.

In everyday practice settings, we do this by making the interaction between healthcare professionals (or teams) and the person (or people) receiving care unilateral and prescriptive. As Figure 4 below seeks to indicate, the exclusive predominance of techno‐scientific knowledge, by focusing the work process on constructing an objective substrate for intervention, restricts the perception of the practical meaning of health attention, marginalizing all interaction coming from the care recipient(s) or reducing it to a subsidiary element of techno‐scientific reasoning and decision‐making by the care provider.

**Figure 5:**
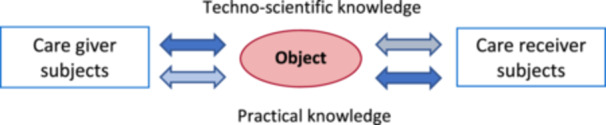
Hermeneutic Interaction Model in Health Practices and Its Praxical Effect. *Source:* Author's creation.

When we enrich the body of scientific knowledge in health practices with contributions from contemporary hermeneutics—such as the kind knowledge proposed by Missel and Birkelund—we certainly bring to health practices a repertoire capable of broadening our perception of health‐illness‐care processes, making professionals more sensitive to the practical aspects involved in their practice. However, this will only break with technicism in the daily routine of health actions if the people to whom these actions are directed have a voice in health work processes, as subjects of praxis that they truly are, so they can effectively participate in the operation of those health work processes (Ayres 2019). The practical experience of all those involved in Care—their knowledge, interests, values, and understanding of their health (or illness)—must jointly construct, alongside the techno‐scientific knowledge of healthcare teams, the ‘what’ and ‘how’ of effective Care, as outlined in Figure 5.

From the perspective of Care, even with varying weights and modes (highlighted by the different shades of the arrows indicating knowledge in the diagram), both healthcare providers and the recipients of their actions are subjects carrying practical and techno‐scientific knowledge relevant to constructing the object/objective of Caring. The diagram in Figure 5 also seeks to represent that, if Care is indeed an experience as Gadamer understands it, then all participants in this encounter will have enriched their practical and techno‐scientific knowledge.

## 5 Final Remarks

In summary, what I hope to have argued is that contemporary hermeneutics also has a contribution to make at the very moment of the clinical act. Care, in the reconstructive sense we have been discussing—that is, aimed at overcoming the techno‐scientism that excludes the praxical dimension from health practices—will depend on an effective ‘*fusion of horizons*’ (Gadamer) between professionals and recipients of health actions, seeking to rely on the ‘*propositional and normative validity*’ (Habermas) of the different type of knowledge of all involved, constructing there, in the act of Caring, a small ‘*hermeneutic arc*’ (Ricoeur), related to broader ones, where the dialectic between explaining (with health sciences) and understanding (with social and human sciences, humanities, and the practical knowledge of people) builds objects and methods of health actions in which theory, technique, and praxis effectively interact, constituting emancipatory subjects and practices. And fortunately, we are not far from this. The practice of health professionals, particularly nursing practice, due to the very nature of its work process, brings us closer to the human, to subjectivity, to affections, to unpredictability on a daily basis. It is therefore necessary that we be attentive to the truths that emerge from this experience and, with our capacity to (re)construct cultural legacies and create the ‘untested feasibility’ (Freire 2005), to seek to promote fruitful encounters between the sciences we produce and the normative horizons that, in an inclusive and fair way, fulfill our highest purposes as individuals and communities.

## Ethics Statement

The author has nothing to report.

## Conflicts of Interest

The author declares no conflicts of interest.

## Data Availability

The author has nothing to report.
